# Cutaneous tuberculosis of the foot clinically mimicking mycetoma: A case report

**DOI:** 10.1002/ccr3.7295

**Published:** 2023-05-04

**Authors:** Ayman Ahmed, Amel Ahmed Hagelnur, Hala Fathi Eltigani, Emmanuel Edwar Siddig

**Affiliations:** ^1^ Swiss Tropical and Public Health Institute (Swiss TPH) Allschwil Switzerland; ^2^ University of Basel Basel Switzerland; ^3^ Tropical Medicine Hospital Khartoum Sudan; ^4^ The Mycetoma Research Center University of Khartoum Khartoum Sudan; ^5^ Unit of Applied Medical Sciences, Faculty of Medical Laboratory Sciences University of Khartoum Khartoum Sudan; ^6^ Department of Medical Microbiology and Infectious Diseases University Medical Center Rotterdam, ErasmusMC Rotterdam The Netherlands; ^7^ Institute of endemic diseases University of Khartoum Khartoum Sudan

**Keywords:** cutaneous, mimics, skin tropical diseases, Sudan, tuberculosis

## Abstract

**Key Clinical Message:**

In a resource‐limited setting such as Sudan, where diagnostic and surveillance capacities are limited and several dermal diseases with similar clinical presentation are endemic, further precautious must taking into account.

**Abstract:**

Cutaneous tuberculosis (CTB) is a rare infection caused by *Mycobacterium tuberculosis*. Atypical clinical presentations of CTB may resemble other skin neglected diseases. For definitive diagnosis, we require a holistic diagnostic approach including clinical examination and deployment of laboratory investigations including microbial culture, histopathological, and molecular examinations of the proper samples per test. In this communication, we report a case of CTB that was initially misidentified clinically as mycetoma.

## 1 INTRODUCTION

*Mycobacterium tuberculosis* infection has different clinical forms that includes pulmonary and extrapulmonary forms.^1^, ^2^ Cutaneous tuberculosis (CTB) is considered a rare form of extrapulmonary tuberculosis which may mimic various other skin dermatoses leading to a misdiagnosis, delay the case management, and eventual increase the disease morbidity.^3^, ^4^ For CTB, the major challenge is the delay in initiating the medical therapy combined with the long duration of the disease, which may become complicated with carcinoma and disfiguring scars.^5^ In this communication, we report a rare presentation of CTB that was initially misdiagnosed as mycetoma due to the strong resemblance. Further clinical examinations by highly trained dermatologist using a well‐established protocol for the differential diagnosis with clear case definition high‐quality ultrasound imaging, and histopathological investigations have aided in making the final diagnosis and improved the case management of the patient.

## 2 CASE REPORT

**FIGURE 1:**
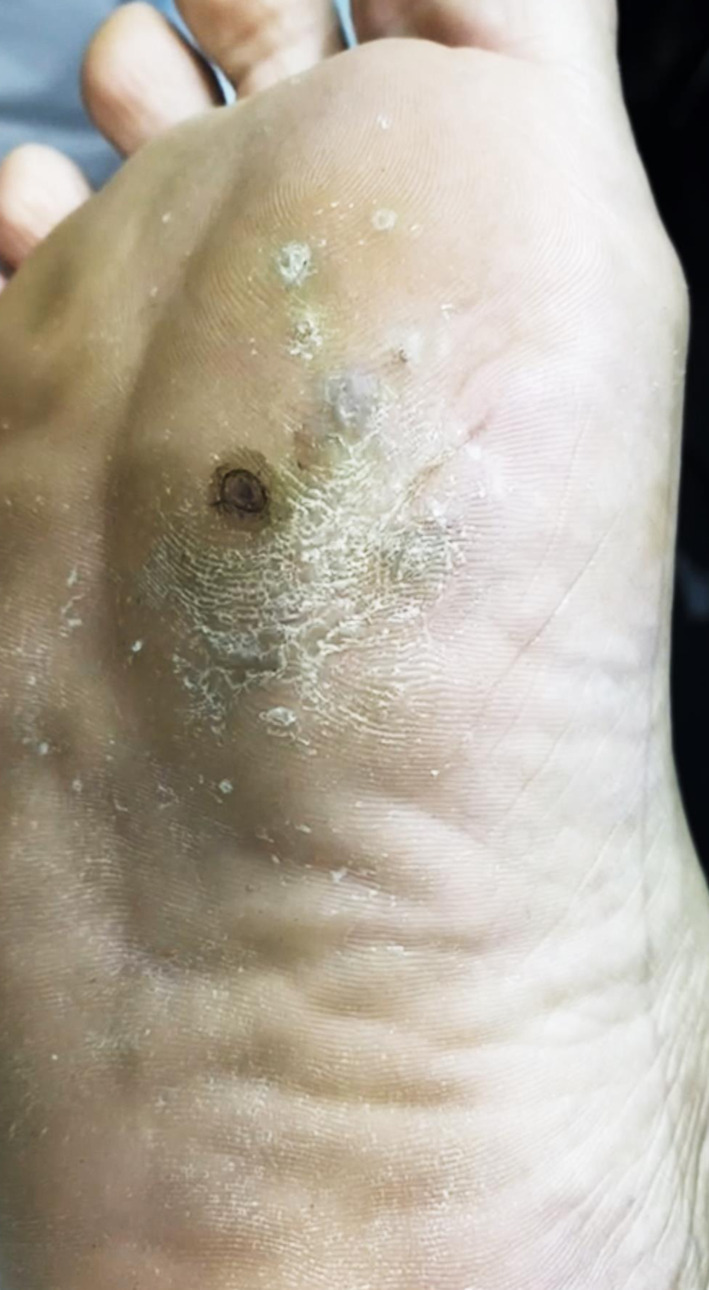
Clinical image showing a lesion over ventral aspect of the mid left foot with multiple discharging sinuses.

A 41‐year‐old male from Kassala state, eastern Sudan, developed a skin lesion over the ventral part of the left foot over the last 7 years (Figure 1). The lesion was initially a small asymptomatic nodule that slowly increased in size, and over time, became painful, itchy, and developed a sinus that discharged foul‐smelling pus. The patient did not give a history of fever, cough, or a previous diagnosis of tuberculosis diagnosis.

On the initial clinical examination at Omderman teaching hospital, he looked well, and there was no pallor, jaundice, or cyanosis. He had normal vital signs and his systemic examinations were within the normal range. Local examination of the effected organ, revealed a lesion in the sole of the left foot that showed a single sinus tract that discharged pus. The other foot was normal and no lesions were found on other parts of the body. The chest x‐ray of the patient was reported as normal. A swab taken from the lesion revealed the growth of *Staphylococcus aureus*. The x‐ray of the foot was normal with no evidence of bone involvement.

Viral screening for human immunodeficiency (HIV) and hepatitis B and C viruses were negative. Liver functions test showed serum bilirubin 0.5 mg/dL, total protein 7.0 g/dL, serum albumin 5.6 g/dL, alkaline phosphatase 75 U/L, aspartate aminotransferase (AST) 19 U/L, and alanine aminotransferase (ALT) 28 U/L. Renal functional test showed a normal value of urea in blood (25 mg/dL) and serum creatinine (0.60 mg/dL). Complete blood count examination showed leucocytosis (12.0 × 10^3^), hemoglobin 13.1 g/dL, and platelets count 397 × 10^3^.

**FIGURE 2:**
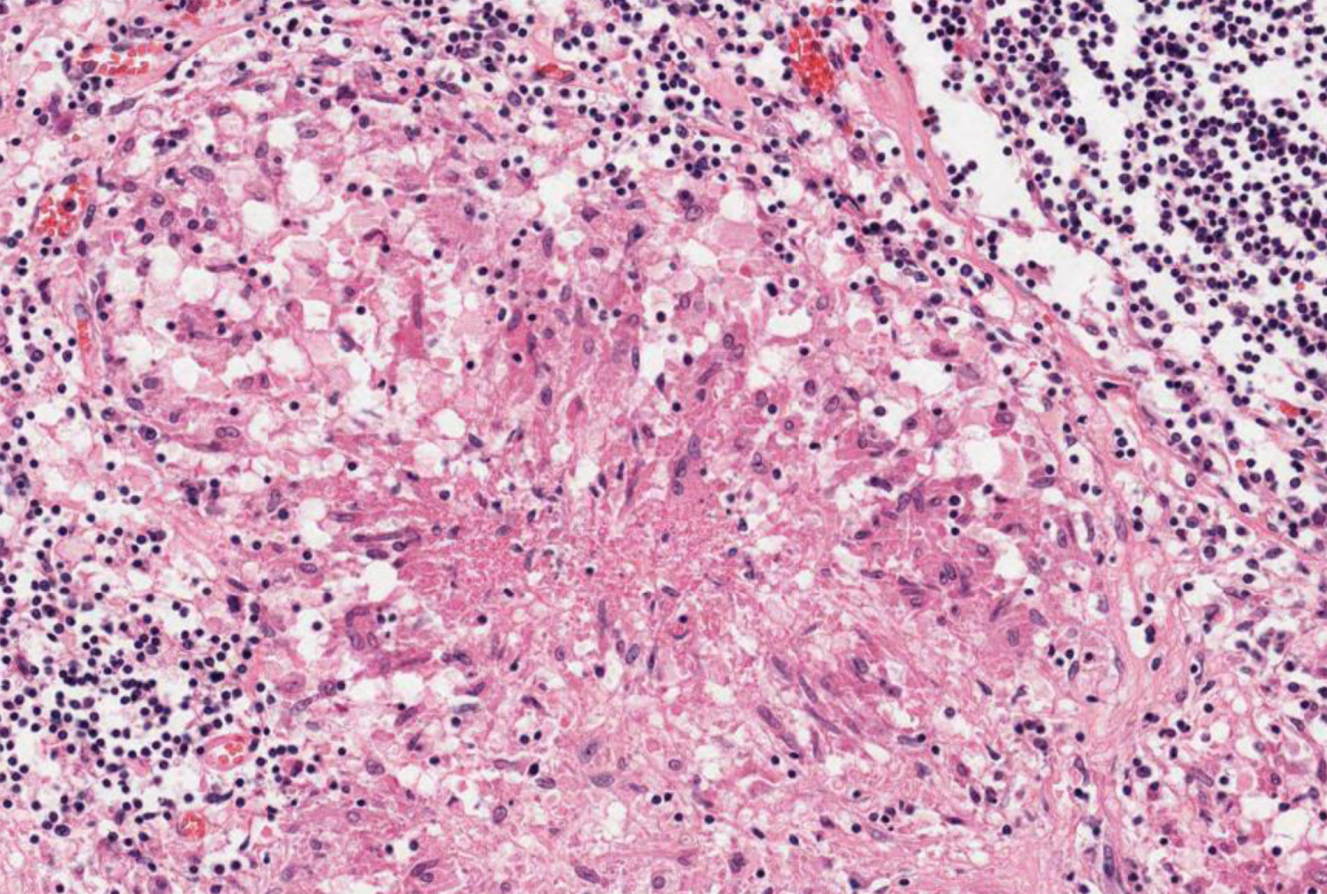
Histological section from the lesion showing epithelioid cells and Langhans giant cells (H&E, ×40).

The lesion was surgically removed under the assumption that it was mycetoma grains. However, the clinical examination of the removed tissue raised some concerns; therefore, a sample was sent to the histopathology laboratory for diagnosis. A paraffin processed tissue block was prepared from the surgical biopsy samples, which measured 2 cm × 1.5 cm. The tissue sample was cut using a rotary microtome, and 2–3 μm sections were obtained. The sections were stained with hematoxylin and eosin stain (H&E) and Ziehl–Neelsen (ZN) staining technique. Microscopic examination showed a granuloma composed of Langhans cells, epithelioid cells, plasma cells, neutrophils, and eosinophils (Figure 2). ZN was negative. A positive culture for *M. tuberculosis* was subsequently obtained from the biopsy specimen of the skin lesion, confirming CTB infection.

A treatment regimen consisted of 2 months of rifampicin, isoniazid, pyrazinamide, and ethambutol was started, followed by rifampicin and isoniazid for another 4 months, with clinical remission.

## 3 DISCUSSION

CTB represents 1%–2% of the total clinical manifestations of tuberculosis infection.^6^ The infection occurs through the invasion of *M. tuberculosis* and *Mycobacterium bovis* bacteria through the skin or following the BCG vaccination.^7^ The infection can be acquired either through inoculation (exogenous form) or through hematogenous spread (endogenous form). Different morphological variants of CTB are existed, which includes lupus vulgaris, scrofuloderma, tuberculosis verrucosa cutis, miliary tuberculosis, and tuberculoid.^6^, ^7^

The diagnosis of CTB is considered challenging and is based on two criteria, either absolute or relative criteria.^1^, ^2^, ^3^, ^4^, ^5^, ^6^, ^7^ The absolute criteria involve identifying *M. tuberculosis* either using culture, molecular tool such as polymerase chain reaction (PCR), or histopathologic examination. The major limitation of diagnosis is the paucibacillary nature of CTB.^5^, ^6^, ^7^ On the other hand, the relative criteria includes detailed history‐taking, lesion assessment, active TB on other organ(s), tuberculoma granuloma on histopathological slides, positive tuberculin test and a good response to TB medications.^6^, ^7^

A major challenge in the diagnosis of CTB is the differential diagnosis as it can mimic numerous infections such as leishmaniasis, leprosy, chromomycosis, sporotrichosis, mycetoma and granulomatous, and verrucous lesion of different origins.^8^, ^9^ Interestingly, mycetoma is an endemic disease in Sudan with more than 7000 reported cases. About 75% of these cases are due to the fungal type (eumycetoma), while the remainder are due to the bacterial type of the disease (actinomycetoma).^10^ Clinically, actinomycetoma mimics CTB,^11^ causing a diagnostic dilemma, and in such a scenario, only biopsy can confirm the diagnosis. In our presented case, the only way to achieve the final diagnosis was through biopsy.

Generally, the challenges faced in diagnosing CTB may be attributed to three characteristics of the disease: (1) the varied clinical presentations of cutaneous TB; (2) the similarities in presentation between cutaneous TB and other skin diseases; and (3) the low bacillary load found in cutaneous TB. Host immunity plays an important role in determining the histopathological picture of cutaneous TB, and the hallmark picture of a typical tuberculoid granuloma which consists of epithelioid cells, lymphocytes, Langhans cell along with caseous necrosis is not present in every patient. Moreover, not all the features are found typically, and their presence and presentation depend on many factors including the host's immune system and ability to organize the granulomatous process. Epithelioid cell granuloma can also be found in other diseases like leprosy, sarcoidosis, and deep fungal infection, which may clinically mimic CTB.^12^ In these cases, a detailed history and a proper clinical examination are needed to rule them out.

In a resource‐limited setting such as Sudan, where diagnostic and surveillance capacities are limited and several dermal diseases with similar clinical presentation are endemic, further precautious must taking into account. Unfortunately, 8 of the 10 skin neglected tropical diseases, namely cutaneous leishmaniasis, post‐kala azar dermal leishmaniasis, leprosy, lymphatic filariasis, mycetoma, onchocerciasis, scabies, and fungal disease are endemic in different regions of the country. Therefore, healthcare providers must be vigilant and maintain a high index of suspicion when investigating suspected cases with disease that have cutaneous manifestations including CTB. Delaying the diagnosis and treatment of diseases including CTB places a physical, psychological, and financial burden on the patients who are already marginalized by their socioeconomic status and lack of access to proper healthcare services. The diagnosis and management of CTB can be improved through educating healthcare providers on the differential diagnosis and how to follow clear case definition in their diagnosis process, and to refer patients to the proper facilities for advanced investigation whenever needed and it was affordable. Furthermore, more investment is needed from the government and their health partners in implementing prevention and control measure in the endemic areas to reduce diseases transmission.

## 4 CONCLUSION

In this communication, we report on a rare case of CTB from Sudan. Healthcare providers in the country should be aware of CTB and it is clinical presentation to improve their differential diagnosis particularly in areas endemic with TB. Particularly that, it can be clinically confused with the co‐endemic diseases in Sudan such as mycetoma.

## AUTHOR CONTRIBUTIONS

**Ayman Ahmed:** Conceptualization; data curation; investigation; methodology; supervision; validation; visualization; writing – original draft; writing – review and editing. **Amel Ahmed Hagelnur:** Investigation; methodology; resources; supervision; writing – review and editing. **Hala Fathi Eltigani:** Investigation; methodology; validation; visualization; writing – review and editing. **Emmanuel Edwar Siddig:** Conceptualization; data curation; formal analysis; investigation; methodology; supervision; validation; visualization; writing – original draft; writing – review and editing.

## FUNDING INFORMATION

None.

## CONFLICT OF INTEREST STATEMENT

The author reports no conflicts of interest in this work.

## ETHICS STATEMENT

A written consent form was obtained from patient.

## CONSENT

Written informed consent was obtained from the patient to publish this report in accordance with the journal's patient consent policy.

## Data Availability

The data that support the findings of this study are available from the corresponding author upon reasonable request.
